# Arthroscopic Fixation of Symptomatic Meso-Type Os Acromiale: Technical Note

**DOI:** 10.1155/2022/1321934

**Published:** 2022-07-25

**Authors:** Quen Oak Tang, Sherif Elnikety

**Affiliations:** ^1^Department of Trauma and Orthopaedics, Watford General Hospital, Watford, UK; ^2^Department of Surgery, College of Medicine and Health Sciences, United Arab Emirate University, UAE

## Abstract

Symptomatic Os acromiale can cause pain, impingement, and reduced range of movement. Disruption of the syndesmosis can result in significant pain and functional impairment; this may occur after trauma. Symptomatic Os acromiale is treated by either excision or fixation. Fixation via open technique is the mainstay of surgical intervention; however, recently, arthroscopic methods were used. In this technical note, we discuss the modification for all arthroscopic Os acromiale fixation; the fixation screws are introduced in anteroposterior fashion, employing the advances in orthopaedic fixation devices. Arthroscopic fixation is not widely adopted, possibly due to availability of implants and perceived difficult learning curve. We report this technique and demonstrate reproducibility with excellent results.

## 1. Background

Os acromiale is a developmental condition which results from failure of fusion of the secondary ossification centre. Four different ossification centres have been identified; failure of fusion of any of them results in mobile Os acromiale [1]. Its prevalence is estimated between 1 and 15% based on different studies. However, it is rarely symptomatic unless it is unstable which may result in impingement symptoms. Symptomatic meso-type Os acromiale can be a significant cause of shoulder dysfunction and pain [2, 3]. Multiple treatment strategies have been tried; no surgical management rarely result in complete cure of the condition due to the structural nature of the condition. Symptomatic meso-type Os acromiale is usually treated surgically; subacromial decompression, excision, or fixation were reported with mixed results, with the majority of surgical techniques open procedures [1, 4, 5]. Due to advances in arthroscopic techniques and surgical implants, we developed all arthroscopic surgical fixation technique for meso-type Os acromiale. The procedure can be successfully performed without the need for X-ray imaging to guide the fixation as traditional fixation technique. Although similar technique was previously described [6], we believe that our technique is easier, less time consuming, and user friendly.

## 2. Case History

A 37-year-old female patient was referred to the orthopaedic department with right shoulder pain. Pain was dull aching in nature over the anterolateral surface of the shoulder, ongoing for at least 12 months. Pain was temporarily relieved by simple analgesia. The patient was otherwise fit and well with no significant comorbidities; she had no trauma and works as an administrator. The patient was diagnosed with subacromial impingement by the general practitioner. She was treated by steroid injection which gave good pain relief for 6 weeks before it gradually increased to its original strength.

Examination of the shoulder revealed positive Hawkin's and Neer's signs of impingement. There was mild weakness of the supraspinatus (pain inhibition) and full range of movement with pain of movements above shoulder level.

Due to the clinical picture and the chronicity of the condition, we elected to proceed with MRI investigation which revealed intact rotator cuff with subacromial bursitis and Os acromiale.

## 3. Technique

**Figure 1:**
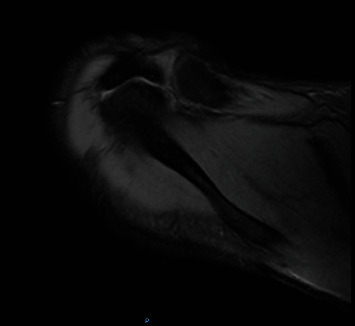
T2-weighted axial magnetic resonance image of meso-type Os acromiale.

**Figure 2:**
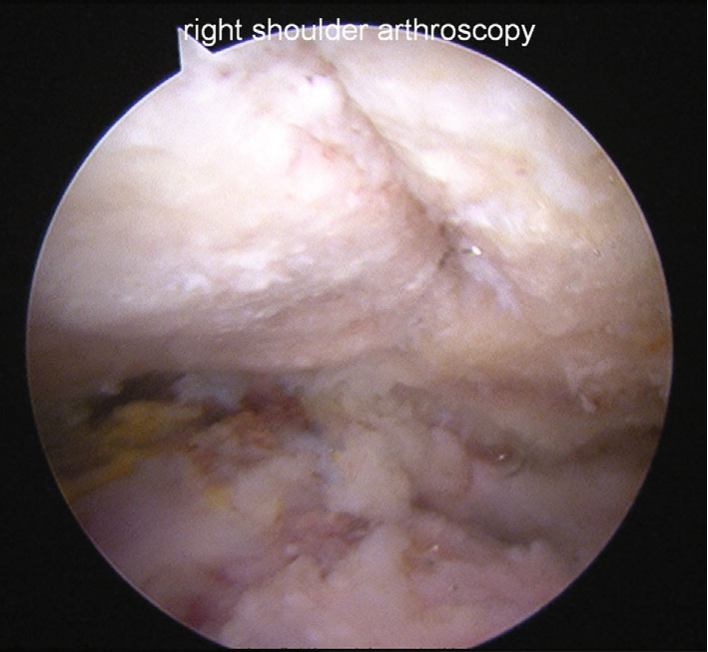
Arthroscopic image of the prepared bony surfaces prior to fixation.

**Figure 3:**
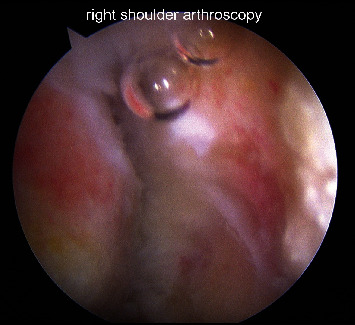
Arthroscopic image of the final position following fixation.

**Figure 4:**
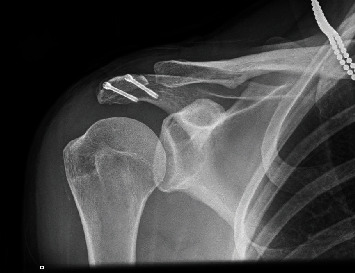
Postoperative radiograph of the final position of the screws.

Clinical and radiological diagnosis was confirmed (Figure 1). Standard beach-chair shoulder arthroscopy was performed and diagnostic intra-articular arthroscopy is done; then, the subacromial space is inspected utilising posterior and posterolateral portals. After excision of the bursa and subacromial soft tissue with radiofrequency ablation, the presence of a mobile meso-type Os acromiale is confirmed. A third lateral portal is made inline with the Os acromiale nonunion. A bone shaver is utilised to debride the nonunion and prepare the bony surfaces for fusion (Figure 2). Two 1.1 mm wires (Medartis AG, Switzerland) are placed percutaneously into the anterior fragment with direct arthroscopic visualisation ensuring that the wires are equally placed into the anterior fragment and in satisfactory position in relation to the anterior fragment. A percutaneous pointed reduction clamp is then placed across the nonunion from a superior direction. Once reduced, the two wires are then advanced across the nonunion site until the posterior cortex is felt by changes in the bony resistance. The wires are measured and appropriately sized self-drilling self-tapping full thread 3.0 mm cannulated compression screws (Medartis AG, Switzerland) are placed percutaneously under constant visualisation to ensure that reduction is maintained (Figure 3). The headless screws are buried within the bone to prevent symptomatic hardware (Figure 4). It is advisable to deduct 2-4 mm of the measured screw length to ensure that the head is fully buried within the bone.

## 4. Postoperative Care

The procedure was done as day case; the patient was followed up at 2 weeks and three months with parallel physiotherapy rehabilitation. The patient was given a broad-arm sling postoperatively. The patient was advised to use the sling for comfort for 4 weeks with active ROM rehabilitation started on day 1 postoperatively. At two weeks appointment, it was found that the arthroscopy wounds have healed, pain was minimal and controlled with simple analgesia when required, and patient was doing active ROM exercises. Postoperative X-ray radiographs were taken at 2 weeks follow-up (Figure 4).

At 3 months follow-up, the patient was found to have full ROM with occasional ache after a period of physical activities and no other symptoms. Patient was given an open follow-up appointment for further 3 months if needed; however, the patient did not request appointment.

## 5. Discussion

Traditionally, Os acromiale fixation is performed via open or arthroscopically assisted approach [5]. Reported results of open fixation were mixed with the risk of secondary procedure for removal of metalwork, while excision surgery runs the risk of deltoid failure [7, 8]. We present a simple arthroscopic technique for percutaneous fixation of symptomatic meso-type Os acromiale. Arthroscopic procedures are widely accepted as the gold standard for shoulder surgery; our technique utilises the shoulder arthroscopy skills and advanced implant designs to perform this procedure all arthroscopically without the need for open incision. This technique has all the benefits of arthroscopy over the open surgery [9]. We believe that our technique is easier and more likely to result in better screw positioning compared to the previously published technique. Absorbable screws are not a necessity, there is a risk of bone cyst formation associated with bioabsorbable screw [10]. Our technique has a shallow learning curve for arthroscopic shoulder surgeons as it utilises pre-existing familiar arthroscopic and orthopaedic skills and benefits from a faster recovery, improved cosmetic result, and minimal violation of the deltoid. Placing the guide wire from anterior to posterior is significantly easier compared to posterior to anterior as this ensures better compression of the Os acromiale fragment, improved screw position in relation to the Os acromiale, and is not affected by variation of the morphology of the acromion, i.e., straight or curved acromion [11]. The use of drill free screws is an additional benefit of our technique. It is less time consuming and it allows improved bone contact with possible increased compression across the nonunion [12]. All suture circulage technique was reported by Guo et al. [1] with reported bony fusion for all cases. Our technique has the advantage of a fully arthroscopic procedure with no need for open intervention. The utilised screws are all buried within the bone which avoids irritation of the suture material underneath the skin, this can be a source of significant irritation to slim patients.

## Abbreviation

ROM: Range of motion.
